# Destabilization of the von Willebrand factor A2 domain under oxidizing conditions investigated by molecular dynamics simulations

**DOI:** 10.1371/journal.pone.0203675

**Published:** 2018-09-17

**Authors:** Gianluca Interlandi

**Affiliations:** Department of Bioengineering, University of Washington, Seattle, WA 98195, United States of America; Jamia Millia Islamia, INDIA

## Abstract

The protein von Willebrand factor (VWF) is key for the adhesion of blood platelets to sites of vascular injury. Recent studies have shown that the release of oxidative agents during inflammation increases the platelet-tethering activity of VWF contributing to a pro-thrombotic state. This has been linked to the oxidation of methionine residues in the A1, A2 and A3 domains of VWF. The A1 domain binds to platelet surface receptors glycoprotein Ib *α* (GpIb*α*). This interaction has been shown to be inhibited under static conditions by the neighboring A2 domain. Tensile force exerted by blood flow unfolds the A2 domain normally leading to its cleavage by the metalloprotease ADAMTS13 preventing pathological thrombus formation. However, oxidizing conditions inhibit proteolysis through ADAMTS13. Here, molecular dynamics simulations tested the hypothesis whether methionine oxidation induced by inflammatory conditions favors unfolding of the A2 domain contributing to the experimentally observed activation of VWF. The results indicate that oxidation of methionine residues located near the C-terminal helix of the A2 domain reduce the force necessary to initiate unfolding. Furthermore, oxidation of methionine residues shifts the thermodynamic equilibrium of the A2 domain fold towards the denatured state. This work suggests a mechanism whereby oxidation reduces the kinetic and thermodynamic stability of the A2 domain removing its inhibitory function on the binding of the A1 domain to GpIb*α*.

## Introduction

The multimeric plasma protein von Willebrand factor (VWF) plays a key role in haemostasis in particular in the presence of rapidly flowing blood like in arteries and arterioles [1, 2]. Shear stress is known to activate VWF, which then recruits blood platelets to the site of vascular injury. Each monomer of VWF consists of a number of domains. The A3 domain of VWF binds to the exposed endothelium while the A1 domain binds to the platelet surface receptor glycoprotein Ib *α* (GpIb*α*). The A2 domain, situated between A1 and A3, contains a proteolytic site that is exposed under shear and cleaved by the metalloprotease ADAMTS13 [3]. This provides a mechanism to downregulate the activity of VWF. Experimental evidence has also suggested that the A2 domain might inhibit the platelet-binding function of A1 when the two domains are bound to each other in the absence of tensile force [4]. Thus, the A2 domain decreases the activity of VWF by exposing its cleavage site under tensile force and likely also by inhibiting the A1 domain under static conditions.

Hyperactivaton of VWF for example due to arterial stenosis can cause pathological thrombus formation. For this reason, VWF has been the target of anti-thrombotic therapies. Importantly, recent experimental evidence indicates that inflammatory conditions are likely to increase the thrombogenic activity of VWF [5, 6]. This is a medically relevant relationship because inflammation has been associated with a pro-thrombotic state [7] and many chronic conditions are often accompanied by systemic inflammation [8]. A key element of inflammation is the production of oxidative agents in particular hypochlorous acid (HOCl). The presence of HOCl has been shown to convert key methionine residues in the A1, A2 and A3 domains of VWF to methionine sulfoxide and to increase the platelet-binding activity of VWF [5]. Furthermore, the A2 domain is rendered uncleavable under oxidizing conditions [6]. It is important to note that these two effects, increased stickiness of VWF and proteolytic resistance of the A2 domain due to oxidation, are independent from each other because ADAMTS13 was not present in the platelet-agglutination assay used to measure VWF activity. Because of these observations, it is essential to study the structure and function of VWF domains under oxidizing conditions. Structural and biophysical studies performed to date on VWF have been done without consideration of the effects of methionine oxidation. This is necessary in order to guide the design of anti-thrombotic drugs that work also under inflammatory conditions.

**Fig 1:**
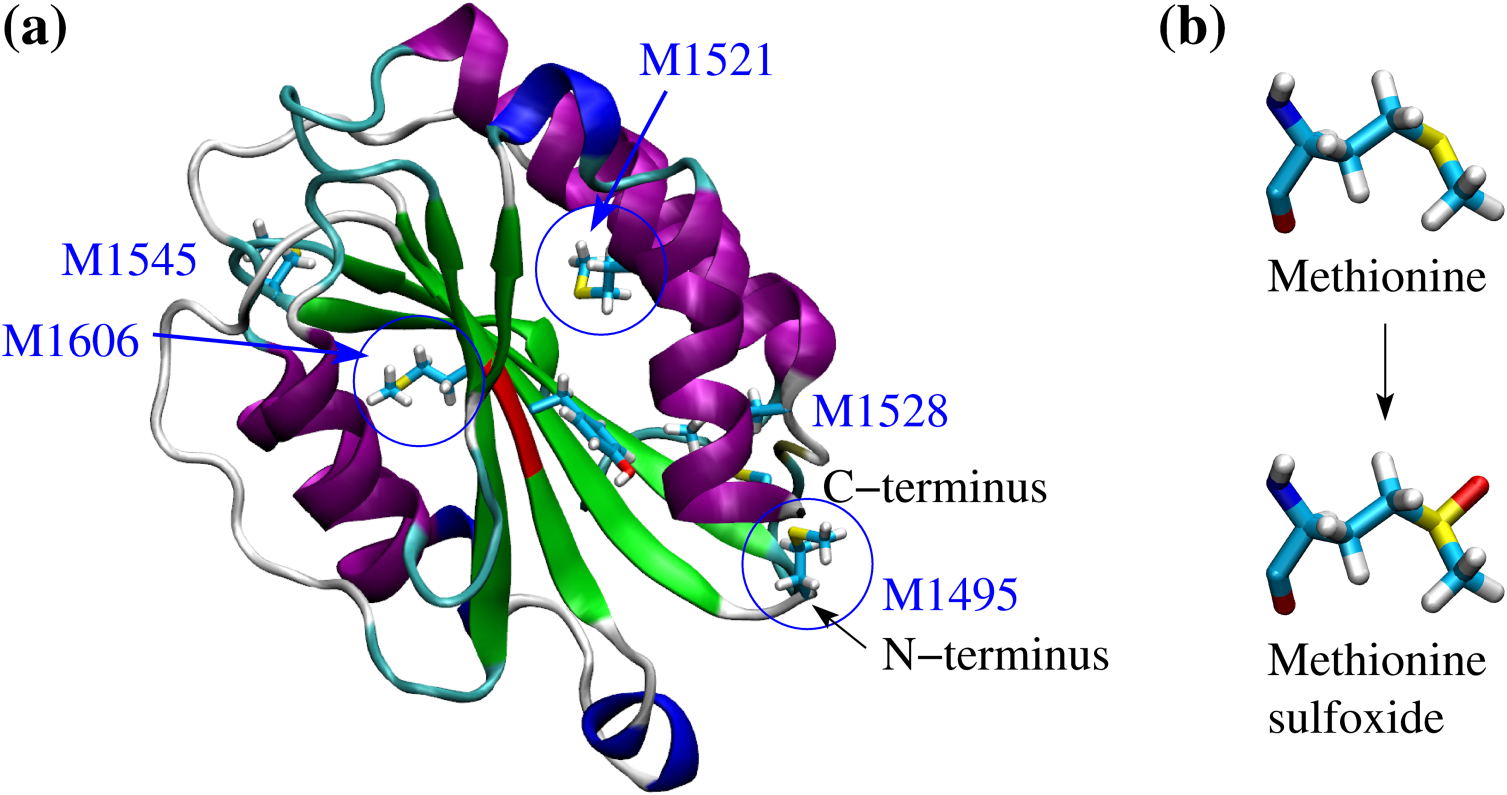
Crystallographic structure of the A2 domain and oxidation of methionine residues. **(a)** Cartoon representation of the A2 domain (PDB code 3GXB). The backbone is colored according to secondary structure elements with *α* helices in purple, 3_10_ helices in blue, *β* strands in green and turns in cyan. The proteolysis site is highlighted in red. The five methionine residues contained within the core of the A2 domain are shown in the stick and ball representation. Blue circles indicate three methionine residues that were determined to be oxidized in mass spectrometry measurements in the presence of HOCl [5]. The other two methionine residues are also likely to be oxidized under the same conditions. **(b)** Conversion pathway from methionine to methionine sulfoxide.

The A1, A2 and A3 domains of VWF consist each of a central *β* sheet surrounded by *α* helices (Fig 1a). However, unlike A1 and A3, the A2 domain does not contain a disulphide bond linking the N- and C-terminii. This makes the A2 domain susceptible to tensile force so that it unfolds under shear exposing its proteolytic site. A previous study performed by us [9], which combined molecular dynamics (MD) simulations with a cleavage assay, investigated mutations located in the C-terminal half of the protein that are clinically associated with type 2A von Willebrand disease, a minor bleeding disorder [10, 11]. The mutations decreased the force necessary to separate the C-terminal *α* helix leading to destabilization of the fold and increased cleavage rate of the A2 domain [9]. Since the A2 domain has been shown to be involved in the regulation of the binding activity of the A1 domain [4] it is plausible that changes in its thermodynamic stability under oxidizing conditions could alter its regulatory function. It has been shown that the presence of HOCl adds an oxygen atom to the sulphur of at least three methionine side chains in A2 converting them to methionine sulfoxide [5] (Fig 1a and 1b). The resulting changes in the structure and force sensitivity of the A2 domain could at least in part explain the increased platelet-binding activity of VWF under oxidizing conditions.

The present work uses molecular dynamics simulations and free energy perturbation (FEP) calculations to study the effect of methionine oxidation on the kinetic and thermodynamic stability of the A2 domain. Since three of the in total five methionine residues in the A2 domain are located in the vicinity of the C-terminal helix it is plausible that their oxidation might destabilize the fold of the A2 domain by facilitating undocking of the C-terminal helix, similarly to a previous study [9]. It is important to note that oxidation hinders proteolysis of the A2 domain. This is because the cleavage site contains a methionine residue, Met^1606^, which is also converted to methionine sulfoxide in the presence of HOCl [6]. Thus it is plausible that under oxidizing conditions unfolding of the A2 domain contributes to the exposure of the A1 domain to blood platelets while cutting through ADAMTS13 is inhibited. The insights gained in this study can guide the design of therapeutic molecules that can for example stabilize the A2 domain only under oxidizing conditions. Such therapeutics would prevent thrombosis when inflammatory conditions persist while still allowing haemostasis under normal conditions.

## Materials and methods

### Initial conformations

The crystallographic structure of the A2 domain with PDB code 3GXB [12] was used to create models where either a single or all methionine residues were oxidized. The models were constructed per homology by replacing the corresponding side chain with methionine sulfoxide and subsequently performing with the program CHARMM [13] 100 steps of steepest descent minimization in vacuo while the positions of all atoms except the mutated residue were kept fixed. It is important to note that although methionine oxidation could in theory result in methionine sulfone, i.e., where two oxygen atoms are covalently bound to the sulphur atom, mass spectrometry data show that oxidizing agents add only one oxygen atom to the methionine residues of the A2 domain, thus resulting in methionine sulfoxide (see supplementary data of reference [5]). The trajectories produced here, where either single or all methionine residues are oxidized, are compared to trajectories obtained with unoxidized A2 domain published in a previous study by us [9].

### General setup of the systems

The MD simulations were performed with the program NAMD [14] using the CHARMM all-hydrogen force field (PARAM22) [15] with the CMAP extension [16, 17] and the TIP3P model of water. The force field parameters for methionine sulfoxide were downloaded from the SwissSidechain website [18]. The different simulations systems are summarized in Table 1. The proteins were inserted into a cubic water box with side length of 80 Å, resulting in a system with in total ca. 50,000 atoms. In the simulations where a tensile force was applied, a rectangular water box was used as described in detail below. Chloride and sodium ions were added to neutralize the system and approximate a salt concentration of 150 mM. The water molecules overlapping with the protein or the ions were removed if the distance between the water oxygen and any atom of the protein or any ion was smaller than 3.1 Å. To avoid finite size effects, periodic boundary conditions were applied. After solvation, the system underwent 500 steps of minimization while the coordinates of the heavy atoms of the protein were held fixed and subsequent 500 steps with no restraints. Each simulation was started with different initial random velocities to ensure that different trajectories were sampled whenever the same primary sequence was simulated. Electrostatic interactions were calculated within a cutoff of 10 Å, while long-range electrostatic effects were taken into account by the Particle Mesh Ewald summation method [19]. Van der Waals interactions were treated with the use of a switch function starting at 8 Å and turning off at 10 Å. The dynamics were integrated with a time step of 2 fs. The covalent bonds involving hydrogens were rigidly constrained by means of the SHAKE algorithm with a tolerance of 10^-8^. Snapshots were saved every 10 ps for trajectory analysis.

**Table 1:** Simulation systems.

Name	Starting structure	Type	Duration [ns]
AllMetO_1,2,3	All 5 Met→Met(O)^a^	300 K^b^	3 x 40
M1495MO_1,2,3	M1495M(O)	300 K^b^	3 x 40
M1521MO_1,2,3	M1521M(O)	300 K^b^	3 x 40
M1528MO_1,2,3	M1528M(O)	300 K^b^	3 x 40
M1606MO_1,2,3	M1606M(O)	300 K^b^	3 x 40
AllMetO_pull_1,2,3	AllMetO_1 (10 ns), AllMetO_2 (10+20 ns)	pulling	3 x 15
M1495MO_pull_1,2,3	M1495MO_1 (10 ns), M1495O_2 (10+20 ns)	pulling	3 x 15
M1521MO_pull_1,2,3	M1521MO_1 (10 ns), M1521O_2 (10+20 ns)	pulling	3 x 15
M1528MO_pull_1,2,3	M1528MO_1 (10 ns), M1528O_2 (10+20 ns)	pulling	3 x 15
M1495_fep_1,2,3	WT_1 (10+20 ns), WT_2 (10 ns)^c^	FEP	3 x 10
M1521_fep_1,2,3	WT_1 (10+20 ns), WT_2 (10 ns)^c^	FEP	3 x 10
M1528_fep_1,2,3	WT_1 (10+20 ns), WT_2 (10 ns)^c^	FEP	3 x 10
M1606_fep_1,2,3	WT_1 (10+20 ns), WT_2 (10 ns)^c^	FEP	3 x 10

^*a*^All five methionine residues at positions 1495, 1521, 1528, 1545 and 1606 were mutated to methionine sulfoxide.

^*b*^These simulations sampled the folded state at room temperature and were not subjected to any perturbation like pulling or FEP.

^*c*^The FEP simulations were started from snapshots sampled during previously published trajectories with the unoxidized wild-type [9]. All simulations were performed in triplicates and labeled with 1, 2 and 3, respectively. The simulations labeled with “300 K” were started after mutating methionine to methionine sulfoxide at the indicated positions in the wild-type structure of the A2 domain with PDB code 3GXB. All other simulations, pulling and FEP, were started from snapshots sampled along the “300 K” runs as indicated.

### Equilibration and sampling of the native state

Before production runs, harmonic constraints were applied to the positions of all heavy atoms of the protein to equilibrate the system at 300 K during a time length of 0.2 ns. To equilibrate the position of atoms around a methionine sulfoxide side chain, harmonic constraints were kept on all heavy atoms except those of the methionine sulfoxide residue and the neighboring amino acids, and equilibration was continued for another 2 ns. After this equilibration phase, the harmonic constraints were released. In order to analyse changes in the native state of the A2 domain upon oxidation, simulations were performed for in total 40 ns each where no external forces were applied (Table 1). The first 10 ns of of unconstrained simulation time were also considered part of the equilibration and were thus not used for the analysis. During the equilibration and in all runs with no tensile force (including the FEP runs described below), the temperature was kept constant at 300 K by using the Langevin thermostat [20] with a damping coefficient of 1 ps^-1^, while the pressure was held constant at 1 atm by applying a pressure piston [21].

### Constant velocity pulling

The simulations with applied tensile force were started from snapshots sampled during the runs with no tensile force described above (see also Table 1). The protein and a bulk layer of 6 Å were removed from the cubic water box and placed into a rectangular water box of 160 Å in the direction of pull and 80 Å in the other two directions. The system was then equilibrated at 300 K as described above. Positional restraints were then applied to the coordinates of the C_*α*_ atom of the N-terminus. The C_*α*_ atom of the C-terminus was attached through a virtual spring with a stiffness constant of 2 kcal/mol to a dummy atom that was pulled at a constant velocity of 5 Å/ns. The initial direction of pull was parallel to the axis through the fixed N-terminal C_*α*_ atom and the pulled atom. As the dummy atom is pulled, the spring extends. Using Hook’s law, the resulting applied tensile force is defined as *F* = Δ*x* * *k*, where Δ*x* is the extension and *k* the stiffness constant of the spring. The tensile force can then be plotted in function of time in order to monitor rupture events. It needs to be noted that from a physical point of view the direction of force is not relevant, because the protein would rotate as a rigid body until the axis through the pulled atoms is aligned parallel to the direction of pull, if this was not the case at the start of the simulation. Similarly, it is generally assumed that if the pulling is performed gently enough the force will propagate through the protein and it will not matter which atom is fixed and which one is pulled. Each pulling simulation was run for 15 ns in order to sample the key events in the unfolding pathway that were identified in a previous study with the A2 domain [9].

### Change in free energy of folding upon oxidation

**Fig 2:**
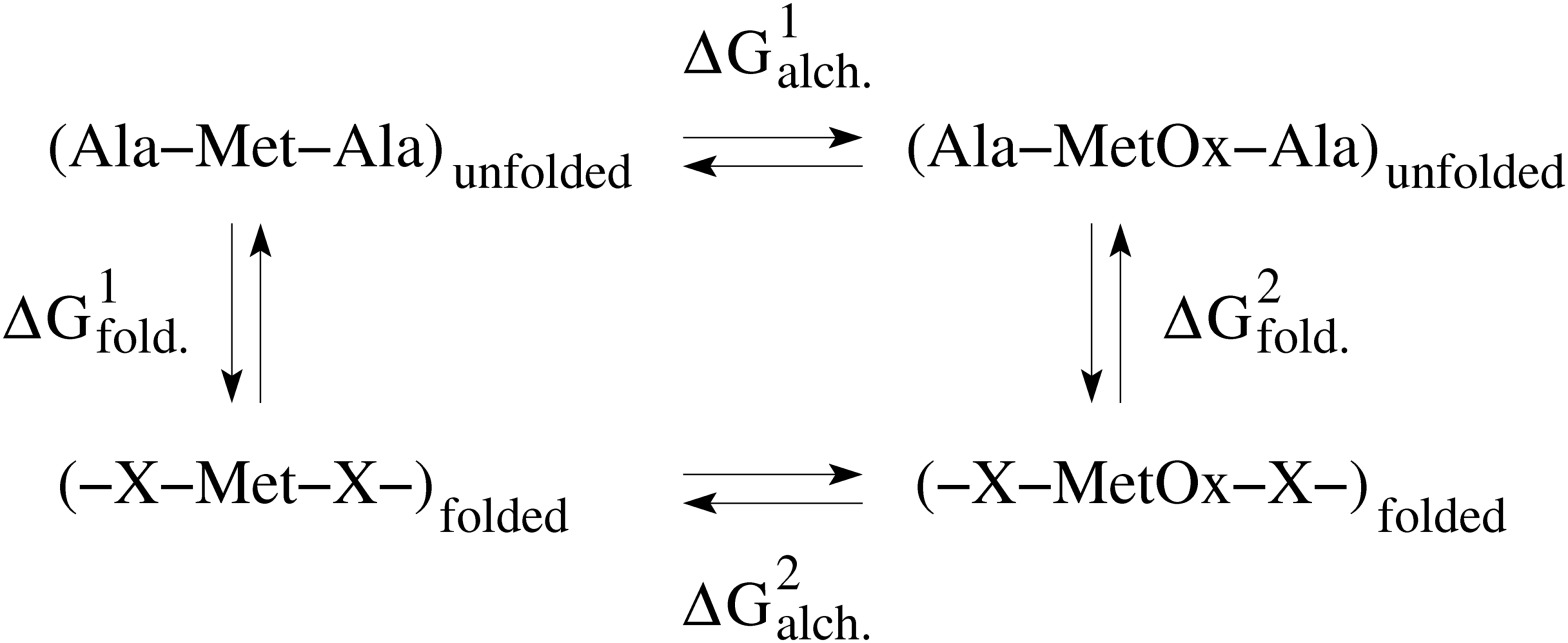
Thermodynamic cycle used to estimate the change in free energy of folding due to the oxidation of a methionine residue. The horizontal arrows correspond to the conversion from methionine to methionine sulfoxide in the folded and unfolded state, respectively. The vertical arrows describe the folding in the unoxidized state and oxidized state, respectively. $\Delta {\text{G}}_{\text{alch}.}^{1,2}$ is calculated as described in the text. The change in free energy of folding can then be derived as follows: $\Delta \Delta \text{G}=\Delta {\text{G}}_{\text{fold}.}^{2}-\Delta {\text{G}}_{\text{fold}.}^{1}=\Delta {\text{G}}_{\text{alch}.}^{2}-\Delta {\text{G}}_{\text{alch}.}^{1}$.

The change in the free energy of folding due to the oxidation of a methionine residue was estimated by making use of alchemical transformations [22] in combination with the thermodynamic cycle [23] represented in Fig 2. The alchemical transformations were performed through FEP calculations [24]. The conformations used to start the computations were taken from previously performed simulations with the A2 domain in the unoxidized state [9]. Each alchemical transformation was performed in the forward and backward direction. In the forward transformation, a methionine side chain is slowly converted to methionine sulfoxide, which contains an oxygen atom covalently bound to the sulphur atom. The conformation achieved in the forward transformation is then used to start a backward transformation where methionine sulfoxide is converted back to methionine. During the process, the amount of work needed for each transformation is calculated. The forward and backward calculations were then combined and a value for the ΔG of the oxidation reaction was obtained using the Bennett’s acceptance ratio method [25] implemented in the ParseFEP plugin of VMD [26]. Each forward and backward transformation was performed for 10 ns during which a parameter λ was varied from 0 (unoxidized methionine) to 1 (methionine sulfoxide) and from 1 to 0, respectively, in time intervals of the length of 0.1 ns for a total of 100 intermediate states. The first half of each time window involved equilibration and the second half data collection. A soft core term was introduced to avoid singularities in the van der Waals potential [27]. Similar ΔG values were obtained when the alchemical transformation was performed for only 1 ns and with time intervals of 0.025 ns (S1 Fig) indicating that the side chain has likely thoroughly sampled its local environment in the nanosecond time scale. Alchemical transformations were performed in the folded and in the unfolded state. The latter was approximated by the situation where a methionine residue is fully solvent exposed, which was accomplished by using a Ala-Met-Ala tripeptide. By considering the thermodynamic cycle (Fig 2), the difference in the free energy of folding upon methionine oxidation can be approximated by the difference between the ΔG values calculated from the alchemical simulations in the folded and unfolded state.

## Results

### Solvent accessibility of methionine residues

Three MD simulations were performed with the A2 domain after replacing all five methionine residues with methionine sulfoxide (Table 1). In addition, sets of three simulations were run after individually mutating each of the four methionine residues, Met^1494^, Met^1521^, Met^1528^ and Met^1606^, to methionine sulfoxide (Table 1). The single point mutation M1545M(O) was omitted because Met^1545^ is fully solvent exposed and thus major conformational changes are not expected (the symbol “(O)” is used here to denote the oxidized state of methionine). The goal is to study any structural and energetic modifications resulting from methionine oxidation on the fold of the A2 domain. It is important to note that methionine residues need to be accessible to solvent in order to be oxidized. Experimental studies show that shear stress increases the likelihood of finding methionine residues of A2 in the oxidized state including those that are buried when the protein is folded [5]. This indicates that the tensile force resulting from shear stress unfolds the A2 domain such that the methionine residues become exposed and accessible to oxidants. It is thinkable that the A2 domain subsequently refolds once shear stress subsides although methionine oxidation might shift the thermodynamic equilibrium toward either the folded or the denatured state. It should also be mentioned that although only three methionine residues in the A2 domain were detected to be oxidized [5] (Fig 1a), it is likely that all five methionine residues are oxidized once VWF is exposed to shear in the presence of HOCl. The analysis presented here was performed on the simulations where all five methionine residues are oxidized. However, similar results were obtained from runs where only one methionine residue was oxidized, respectively, and are reported in the Supporting Information. The results are compared to previously performed simulations with the unoxidized wild-type of the A2 domain [9].

**Fig 3:**
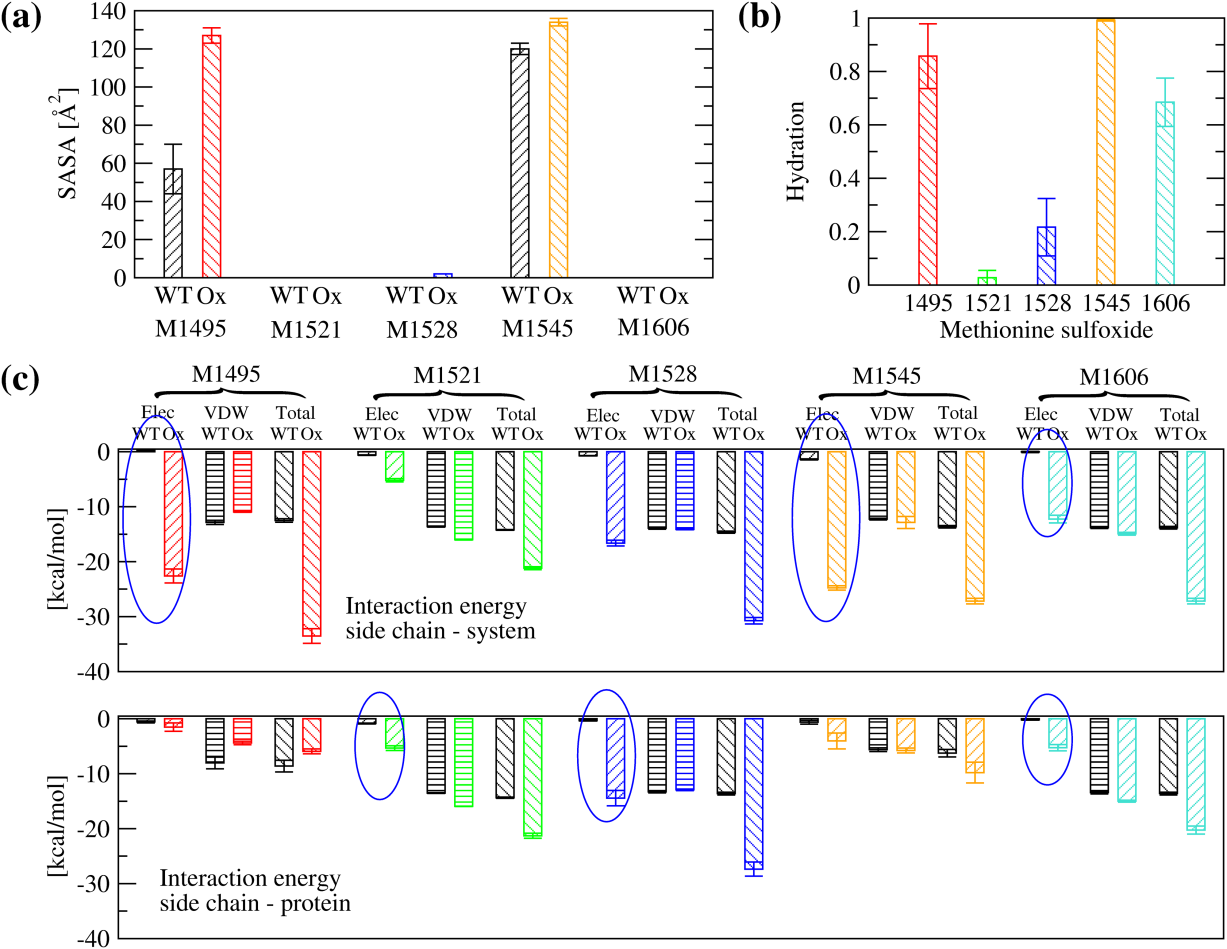
Solvent accessibility and energetic analysis of methionine residues. **(a)** SASA of methionine residues in the unoxidized (called here wild-type, WT) and the oxidized (Ox) state. **(b)** Fraction of frames where a methionine sulfoxide forms at least one hydrogen bond with water molecules. A hydrogen bond was defined using a H…O distance cutoff of 2.7 Å and a O-H…O angle cutoff of 120°. **(c)** Interaction energy between a methionine side chain and (top) the rest of the system or (bottom) the rest of the protein, respectively. Blue circles highlight whether the change in electrostatic energy upon oxidation is due to the interaction with the solvent or the rest of the protein, or both. The simulations of the oxidized state were performed with all five methionine residues converted to methionine sulfoxide (runs AllMetO_1,2,3 in Table 1) and compared in (a) and (c) to previously published runs with the unoxidized wild-type A2 domain [9]. The represented values are averages over three simulations while error bars denote standard errors of the mean.

**Fig 4:**
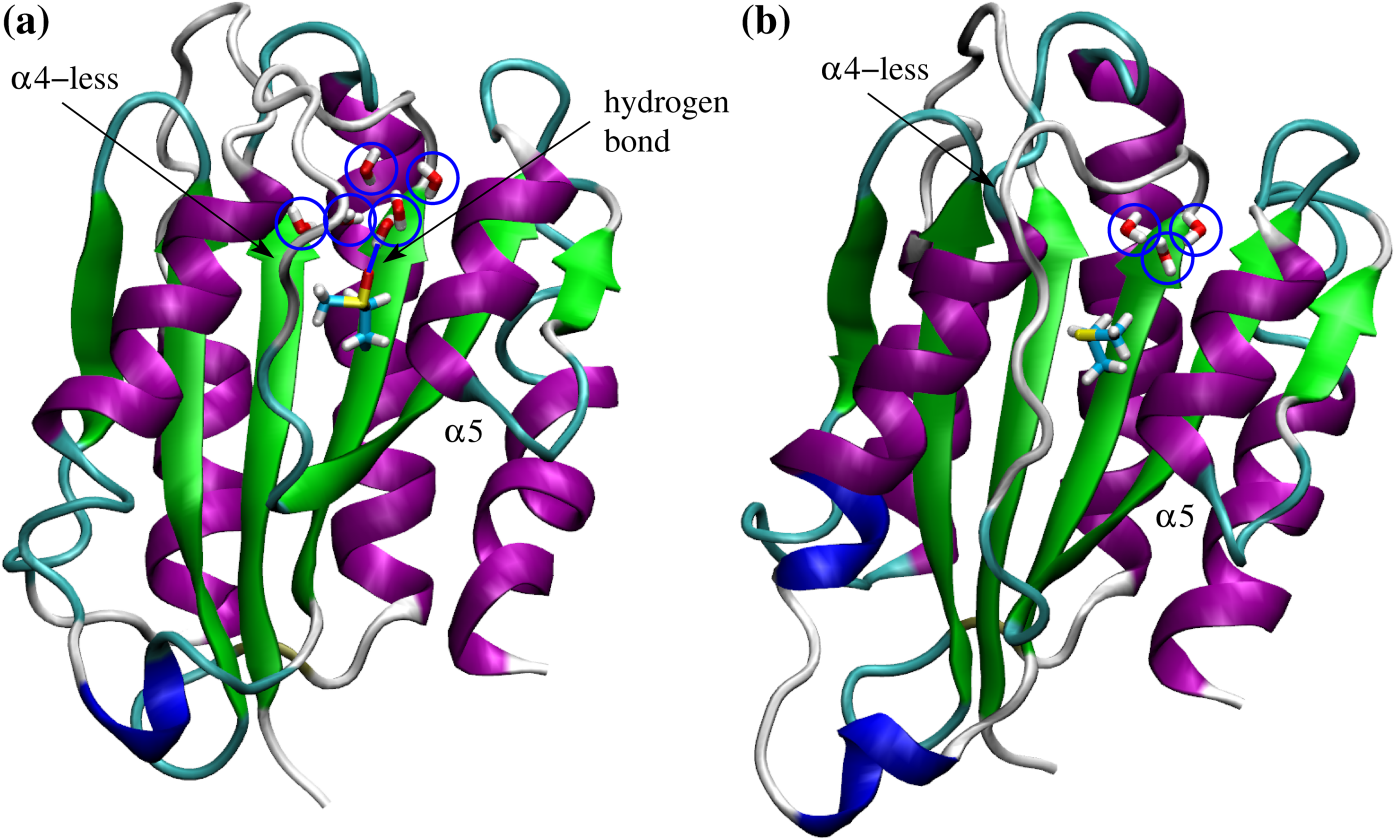
Water channel leading to Met^1606^. **(a)** Example of water molecules (highlighted by blue circles) that have penetrated into the interior of the A2 domain and hydrate Met(O)^1606^. Displayed is the simulation frame after 20 ns in run AllMetO_1 (Table 1). Water molecules with any atom within 6 Å from any atom of Met(O)^1606^ are shown and one hydrogen bond between a water molecule and the sulfinyl group of Met(O)^1606^ is highlighted. Indicated are also the *α*4-less loop and the *α*5 helix. Water molecules are observed in the simulations to penetrate through a gap between the *α*4-less loop and the *α*5 helix. **(b)** Water molecules within 6 Å of Met^1606^ present in the crystallographic structure of the A2 domain (PDB code 3GXB).

Analysis of the solvent accessible surface area (SASA) shows that as expected Met^1545^ has a significant portion of its side chain solvent exposed in both the unoxidized and the oxidized state (Fig 3a). On the other hand, Met^1495^ is partially solvent exposed when unoxidized but in its oxidized state it presents a similar SASA as Met(O)^1545^ (Fig 3a and S2a Fig). All other three methionine residues are relatively buried whether oxidized or not (Fig 3a and S2a Fig). However, analysis of hydration shows that Met(O)^1606^ forms hydrogen bonds with water molecules (Fig 3b and S2b Fig) although it is buried inside the protein (Fig 3a and S2a Fig). Visual analysis of the trajectories reveals the presence of a water channel between an unstructured loop (called *α*4-less loop) and a neighboring helix (called *α*5 per analogy to the helices of the A1 domain [9]), which allows penetration of water molecules that reach the side chain of Met(O)^1606^ (Fig 4a). Interestingly, the crystallographic structure of the A2 domain also displays internal water molecules in the vicinity of Met^1606^ in the crystalline state (Fig 4b). Smaller amounts of hydration are observed also for Met(O)^1528^ and Met(O)^1521^ (Fig 3b and S2b Fig).

The differential solvent accessibility and hydration of methionine residues partly explains differences in the electrostatic interaction energy between the methionine residues and the rest of the system or the rest of the protein (Fig 3c and S2c Fig). The side chains of Met(O)^1495^ and Met(O)^1545^ have a large electrostatic interaction energy due to their accessibility to solvent molecules (Fig 3c). Because of the water channel (Fig 4), the side chain of Met(O)^1606^ also displays a modest amount of electrostatic interaction energy with the solvent (larger values when calculated with the rest of the system than with the rest of the protein, Fig 3c and S2c Fig). On the other hand, Met(O)^1521^ and Met(O)^1528^ interact mainly with polar groups in the interior of the protein (similar values when calculated with the rest of the system or the rest of the protein, Fig 3c and S2c Fig). For these reasons, it is plausible that buried methionine residues in the A2 domain might be accessible to oxidants also in the absence of tensile force. This is consistent with mass spectrometry measurements where Met^1521^ and Met^1606^ are detected to be oxidized under static conditions albeit at a lower rate than under shear [5].

### Unfolding under tensile force

**Fig 5:**
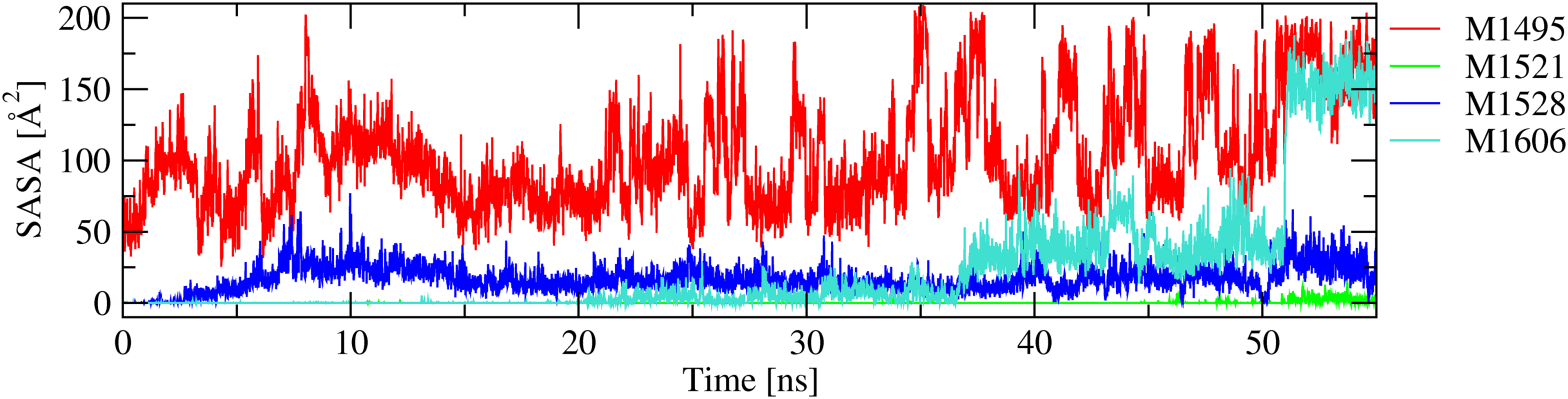
Solvent exposure of methionine residues during unfolding. The coordinates of the N-terminal C_*α*_ atom were held fixed while the C-terminal C_*α*_ atom was pulled at constant velocity. The time series of Met^1545^ is not shown because this residue is already solvent exposed in the folded state. Presented here is an analysis of a previously published trajectory (run WT_pull_1) [9]. Similar results were observed in two replicate simulations shown in S3 and S4 Figs.

The A2 domain is known to unfold under tensile force generated by shear forces in blood [9, 28]. It is likely that this stretching process leads to exposure of methionine residues and their subsequent increased rate of oxidation. Fig 5 shows a sequential exposure of methionine residues during unfolding of the A2 domain induced by tensile force in previously published simulations with unoxidized wild-type [9] that were re-analysed for this manuscript. The side chain of Met^1495^ is the first to become fully exposed, followed by Met^1528^, Met^1521^ and finally Met^1606^, which is located in the proteolytic site. Although the process of oxidation itself cannot be simulated in classic MD simulations, it is plausible that solvent exposure of methionine residues increases their likelihood of becoming oxidized, making them hydrophilic and subsequently facilitating further unfolding.

**Fig 6:**
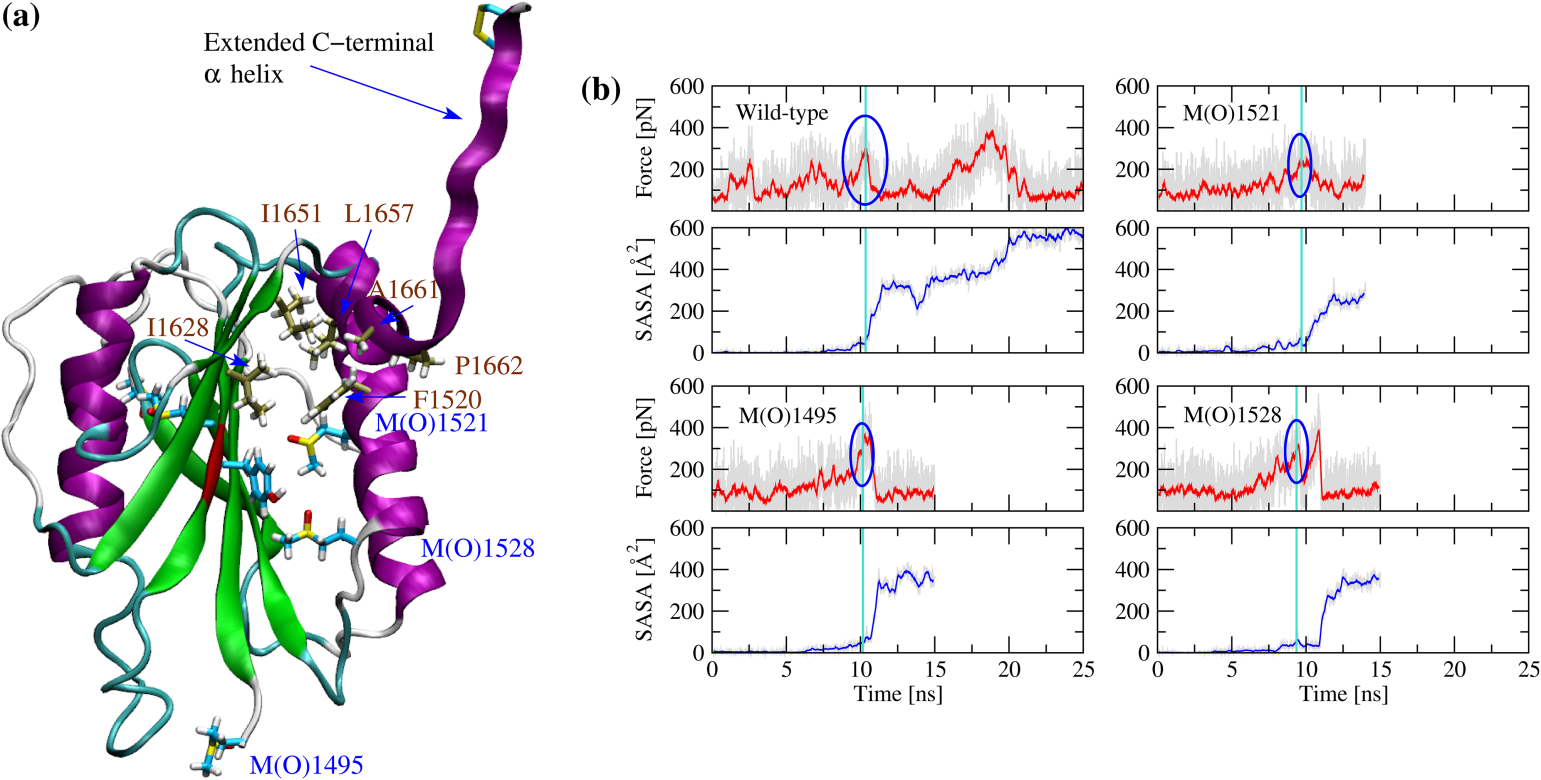
Pulling simulations and undocking of the C-terminal *α* helix. **(a)** Snapshot saved after ca. 10 ns of a pulling run where all methionine residues are oxidized (MetAllO_pull_1). The methionine sulfoxide residues located in the vicinity of the C-terminal *α* helix are labeled in blue. The residues forming the C-terminal hydrophobic core are labeled in brown and their carbon atoms are colored in tan for distinction. The backbone of the proteolytic site is colored in red. This snapshot represents the event where the C-terminal hydrophobic core becomes exposed to solvent and the C-terminal *α* helix separates from the rest of the protein. **(b)** Time series of the applied force and SASA of the C-terminal hydrophobic core (first set of runs). Blue circles highlight the force peak at which the C-terminal helix completely separates from the rest of the protein. The magnitude of the force peak was determined by identifying the time point when the SASA of the C-terminal hydrophobic core exceeds 50 Å^2^ (indicated by a vertical cyan line) and searching for the highest value of the force within a 400 ps time window. Running averages over 20 ps are indicated in red for the force and in blue for the SASA, respectively. The time series with the unoxidized with-type are taken from a previously published study (WT_pull_1) [9]. Replicas of the simulations presented in these plots and pulling simulations with all methionine residues oxidized are presented in S5 to S7 Figs.

**Fig 7:**
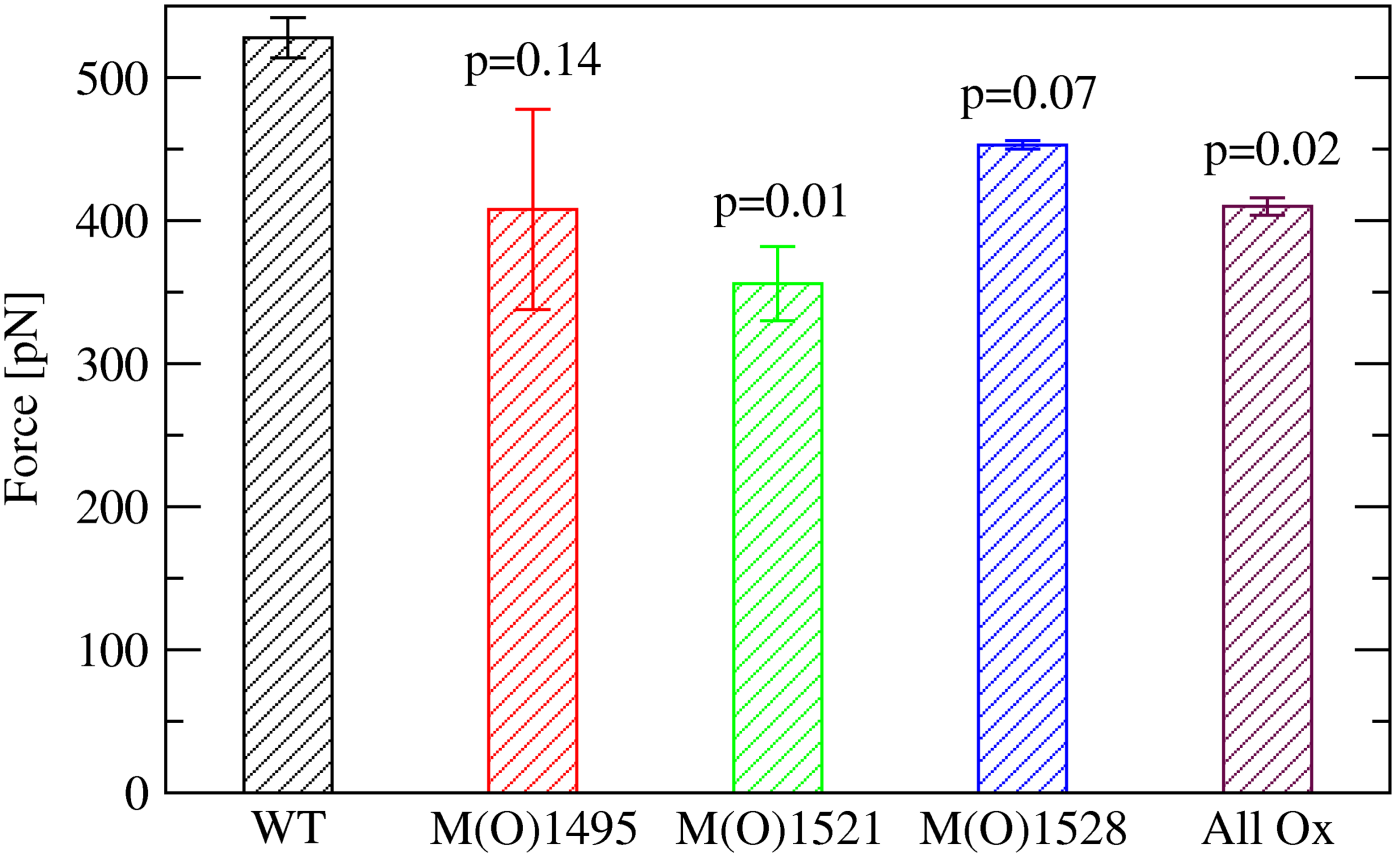
Mean and standard error of the mean of the force peaks corresponding to the solvent exposure of the C-terminal hydrophobic core. The values are averaged over three simulations performed with either the indicated methionine or all methionine residues oxidized. Reported are the p-values from a one-tailed student t-test. A value below 0.05 was considered statistically significant whereas a value between 0.05 and 0.10 was said to be marginally statistically significant.

In order to understand whether and how methionine oxidation facilitates unfolding of the A2 domain, simulations were performed in the presence of tensile force. As in a previous study by us [9], the N-terminal C_*α*_ atom was held fixed while the C-terminal C_*α*_ atom was attached through a virtual spring to a dummy atom that was pulled at constant velocity. This setup allows monitoring the magnitude of the force applied to the C-terminal C_*α*_ atom in order to identify rupture events, i.e., events after which the force suddenly drops. A previous study performed with unoxidized A2 domain revealed that unfolding begins with undocking of the C-terminal *α* helix [9]. This event is characterized by the solvent exposure of hydrophobic residues buried by two N-terminal coils of the C-terminal *α* helix and referred to as the C-terminal hydrophobic core (Fig 6a). Mutations located in the vicinity of the C-terminal helix were shown to lower the force necessary to expose the C-terminal hydrophobic core and lead to an overall destabilization of the A2 domain [9]. Since three methionine residues are located in the vicinity of the C-terminal *α* helix (Fig 6a), it is likely that their oxidation could also facilitate unfolding under tensile force. For this reason, simulations were run with either Met^1495^, Met^1521^ or Met^1528^ individually converted to methionine sulfoxide, or with all five methionine residues oxidized. All simulations were performed in triplicates to obtain statistics and the results were compared to previously published runs with unoxidized A2 domain [9]. Analysis of the trajectories shows that the force necessary to undock the C-terminal *α* helix and expose the C-terminal hydrophobic core to the solvent is indeed lower when at least one of the methionine residues located in its vicinity is oxidized than in the unoxidized state of the A2 domain (Figs 6b and 7). We can conclude that tensile force leads to the sequential solvent exposure of methionine residues, and as these become oxidized hydration of the interior of the protein is increased facilitating the separation of the C-terminal *α* helix.

### Analysis of thermodynamic stability

**Fig 8:**
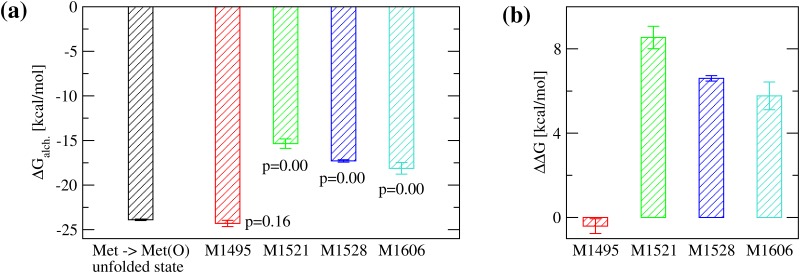
Estimate for the change in free energy of folding upon oxidation. **(a)** Calculated ΔG_alch._ for the transformation from methionine to methionine sulfoxide using FEP. The calculations were done with the tri-peptide Ala-Met-Ala to mimic the unfolded state (black bar) and for each methionine residue in the folded state of the A2 domain that is at least partially buried (colored bars). The residue Met^1545^ was excluded because it is completely solvent exposed. The p-values for the difference in ΔG_alch._ between folded and unfolded state were calculated from a one-tailed student t-test. **(b)** The estimated ΔΔG of folding due to the conversion of an individual methionine residue to methionine sulfoxide (see “Materials and methods” and Fig 2 for details about how it was calculated). A positive value indicates that oxidation of a specific methionine residue is thermodynamically unfavorable for folding. The reported values are averages over three simulations while error bars denote standard errors of the mean.

It is likely that when tensile force across the A2 domain subsides, the protein refolds into a similar structure as the unoxidized state even if it contains methionine sulfoxide residues. However, because of the burial of sulfinyl groups, which are hydrophilic, it is also thinkable that the thermodynamic equilibrium shifts towards the unfolded state. Experimentally, changes in free energy of folding of a protein due to a single-point mutation are often measured through denaturation experiments. Therein, the Gibb’s free energy difference between folded and denatured state, ΔG, is determined in both the wild-type and the engineered mutant and then subtracted from each other yielding ΔΔG. Estimating the ΔG of folding *in silico* would be computationally very expensive because it would require an exhaustive sampling of the free energy landscape. A simpler computational approach is the use of alchemical transformations where a side chain is mutated in the folded and in the unfolded state, both times calculating the amount of work needed to do so, and then by using the thermodynamic cycle to obtain an estimate of ΔΔG (Fig 2). Here, this method is applied to analyse the conversion from methionine to methionine sulfoxide. Analysis of the results shows that indeed oxidation of buried methionine residues is unfavorable for the folding of the A2 domain (Fig 8). This indicates that once the methionine residues in the A2 domain become oxidized, the thermodynamic equilibrium shifts away from the native state and the protein is more likely to be found in the denatured state than before oxidation.

## Discussion

The inflammatory response following vessel wall endothelial damage is part of a natural defense mechanism, which involves recruiting inflammatory cells that attack micro-organisms and endotoxins [29]. However, in particular chronic inflammation has been associated with a pro-thrombotic state [7]. The oxidizing agent HOCl produced during the inflammatory response has been shown to increase the platelet-tethering activity of VWF and to convert methionine residues in the A1, A2 and A3 domains to methionine sulfoxide [5]. Since the A2 domain normally has an inhibitory function on the A1 domain [30], it is plausible that oxidation alters the A2 domain fold in such a way that it no longer masks the A1 domain binding site to platelets. The present manuscript explored this question using MD simulations.

**Fig 9:**
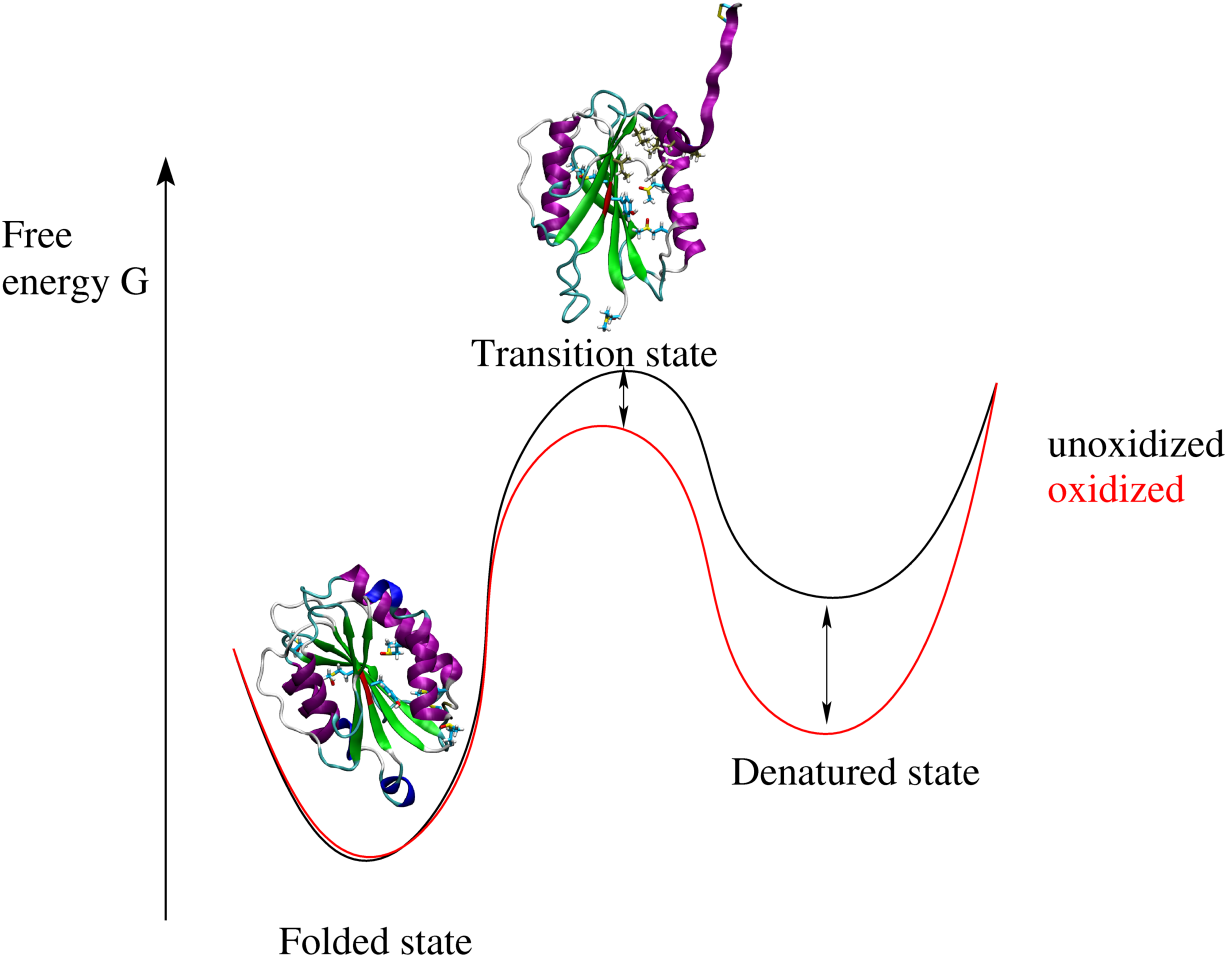
Cartoon illustrating the effects of methionine oxidation on the free energy landscape of the A2 domain.

The simulation results indicate that oxidation alters the free energy landscape of the A2 domain. This is illustrated by a cartoon in Fig 9. Experimentally, the presence of shear is known to increase the rate of oxidation of buried methionine residues compared to static conditions [5]. The pulling simulations presented here suggest that oxidation of methionine residues located near the C-terminal helix can facilitate unfolding under tensile force reducing the free energy barrier between folded and transition state (Fig 9). Oxidation of buried methionine residues can either occur under static conditions due to local flexibility allowing for solvent penetration (Fig 3b) or to the sequential exposure to solvent while the A2 domain unfolds under tensile force (Fig 5). Furthermore, alchemical calculations point out that the free energy difference between the folded and the denatured state shrinks upon oxidation (Fig 9). Thus, the thermodynamic equilibrium is shifted towards the unfolded state, i.e., the A2 domain is more likely to be found in the denatured state under oxidizing conditions (Fig 9).

Under normal conditions, destabilization of the A2 domain, induced either by tensile force or mutations, leads to an increased cleavage by ADAMTS13. This has been shown for example in the case of mutations that are clinically linked to the bleeding disorder type 2A von Willebrand disease [9]. However, under oxidizing conditions, the A2 domain can no longer be cleaved by the metalloprotease [6]. This has been linked to the conversion of Met^1606^ to methionine sulfoxide preventing recognition of the scissile bond [6]. Thus, despite oxidation facilitates denaturation of the A2 domain, the thrombogenic activity of VWF cannot be reduced since cleavage through ADAMTS13 is hindered. On the contrary, it is likely that the instability of the oxidized A2 domain contributes to the increased platelet binding activity observed under oxidizing conditions [5]. This can happen through the following two mechanisms. One plausible mechanism is that destabilization of the A2 domain due to methionine oxidation is likely to interfere with its masking function of the A1 domain [30] facilitating binding to blood platelets. Another possible mechanism is an increase in the propensity of VWF to self-assemble and form fibers. Since the A2 domain unfolds under shear and VWF self-assembly has also been shown to be driven by shear [31] it is plausible that the A2 domain in its unfolded state could mediate the lateral attachment of VWF strands into fibers.

The relationship between inflammation and the thrombotic function of VWF is important because elevated levels of VWF have been observed in patients suffering of inflammatory conditions, such as HELLP syndrome (hemolysis, elevated liver enzymes and low platelets), antiphospholipid syndrome and sepsis [32]. Thus, it is wishful to develop therapeutics that can minimize the risk of thrombosis under inflammatory conditions while at the same time allow haemostasis in case of vascular injury. The results from this manuscript suggest that since unfolding starts with the C-terminal helix, a molecule could be designed that binds between the C-terminal helix and the rest of the A2 domain only when the methionine residues are oxidized. Such a molecule would prevent the A2 domain from unfolding reducing the binding strength of the A1 domain to platelets.

A recent single molecule force spectroscopy study indicated that the A2 domain can exert its inhibitory function only when the disulfide bond between two C-terminal vicinal cysteine residues is reduced [33]. Although the simulations here were performed with the disulfide bond formed, the destabilization induced by methionine oxidation most likely occurs also when the disulfide bond is reduced because its redox state has been found experimentally to not alter the structure of the A2 domain [34].

Of all three A domains of VWF, the A2 domain is the most sensitive to tensile force because it does not have a disulfide bond linking the terminii, thus facilitating oxidation of methionine residues under shear. However, mass spectrometry measurements show that buried methionine residues in the A1 domain also have an increased rate of oxidation under shear, although not as pronounced as for the A2 domain [5]. Two of the methionine residues are located in an area of the A1 domain where congenital mutations are commonly found that are associated with type 2B von Willebrand disease, a bleeding disorder where VWF binds strongly to platelets clearing them from plasma. A previous study by us indicated that von Willebrand disease type 2B mutations may act by displacing the inhibitory N-terminal linker of the A1 domain activating binding [35]. It is plausible that oxidation might induce a similar effect on the A1 domain, which would be complementary to the effects due to oxidation of the A2 domain. It is also thinkable that oxidation of exposed methionine residues in the A1 domain located distally to the N-terminal linker region could facilitate separation from the A2 domain and thus exposure of the platelet-binding site. Overall, there could be multiple mechanisms how oxidation activates VWF just like there are multiple mechanisms how shear promotes binding of VWF to platelets. Studying additional mechanisms how oxidation activates VWF besides facilitating A2 domain unfolding will be the focus of future studies.

A noteworthy observation from a structural perspective is the correlation between the SASA of a methionine residue and its change in folding free energy upon oxidation. Oxidation of buried methionine residues, such as Met^1521^ and Met^1528^, causes a relatively large local destabilization. On the other hand, the free energy of folding appears unaffected when a solvent exposed methionine residue, such as Met^1495^, is oxidized. This is consistent with an increase of the hydrophilicity of a methionine residue once it gains a sulfinyl group conferring to the otherwise rather hydrophobic methionine side chain the ability to engage in hydrogen bonds. The work presented here is the first report to date where an alchemical free energy method has been applied to an amino acid modification due to environmental factors, like oxidation, rather than an engineered mutation. It would be interesting to apply the method used here to other examples where methionine oxidation is suspected to play a role in protein stability or function. In conclusion, the present study suggests that the experimentally observed activation of VWF under oxidizing conditions [5] is at least in part due to destabilization of the A2 domain, which leads to exposure of the platelet-binding site in the A1 domain. Furthermore, a free energy method is presented to study the effects of methionine oxidation on the thermodynamic stability of proteins.

## Supporting information

S1 Fig Comparison between ΔG_alch._ calculated through FEP simulations run for 1 ns vs 10 ns. The reported values are averages over three simulations while error bars denote standard errors of the mean.
(TIF)

S2 Fig Solvent accessibility and energetic analysis of methionine residues. **(a)** SASA of methionine residues in the unoxidized (called here wild-type, WT) and the oxidized (Ox) state. **(b)** Fraction of frames where a methionine sulfoxide forms at least one hydrogen bond with water molecules. **(c)** Interaction energy between a methionine side chain and (top) the rest of the system or (bottom) the rest of the protein, respectively. Blue circles highlight significant electrostatic interactions between a residue and the solvent or the rest of the protein, respectively. Simulations were performed with the respective methionine residue in the oxidized state (runs M1495MO_1,2,3, M1521MO_1,2,3, M1528MO_1,2,3 and M1606MO_1,2,3 in Table 1) and compared in (a) and (c) to previously published runs with the unoxidized wild-type A2 domain [9]. The represented values are averages over three simulations while error bars denote standard errors of the mean.
(TIF)

S3 Fig Solvent exposure of methionine residues during unfolding. Presented here is an analysis of a previously published trajectory (run WT_pull_2) [9].
(TIF)

S4 Fig Solvent exposure of methionine residues during unfolding. Presented here is an analysis of a previously published trajectory (run WT_pull_3) [9].
(TIF)

S5 Fig Time series of the applied force and SASA of the C-terminal hydrophobic core (second set of runs). The vertical cyan line indicates the time point where the SASA exceeds 50 Å^2^. The blue circles highlight the corresponding peak in force. Running averages over 20 ps are indicated in red for the force and in blue for the SASA, respectively. The time series with the unoxidized with-type are taken from a previously published study (WT_pull_2) [9].
(TIF)

S6 Fig Time series of the applied force and SASA of the C-terminal hydrophobic core (third set of runs). The vertical cyan line indicates the time point where the SASA exceeds 50 Å^2^. The blue circles highlight the corresponding peak in force. Running averages over 20 ps are indicated in red for the force and in blue for the SASA, respectively. The time series with the unoxidized with-type are taken from a previously published study (WT_pull_3) [9].
(TIF)

S7 Fig Time series of the applied force and SASA of the C-terminal hydrophobic core (runs with all methionine residues oxidized). The vertical cyan line indicates the time point where the SASA exceeds 50 Å^2^. The blue circles highlight the corresponding peak in force. Running averages over 20 ps are indicated in red for the force and in blue for the SASA, respectively. The time series with the unoxidized with-type are taken from a previously published study (WT_pull_1) [9].
(TIF)
