# Supplementary figures and images for: Destabilization of the von Willebrand factor A2 domain under oxidizing conditions investigated by molecular dynamics simulations
Source: PLoS One. 2018 Sep 17;13(9):e0203675. doi: 10.1371/journal.pone.0203675 (PMC6141083; doi:10.1371/journal.pone.0203675)

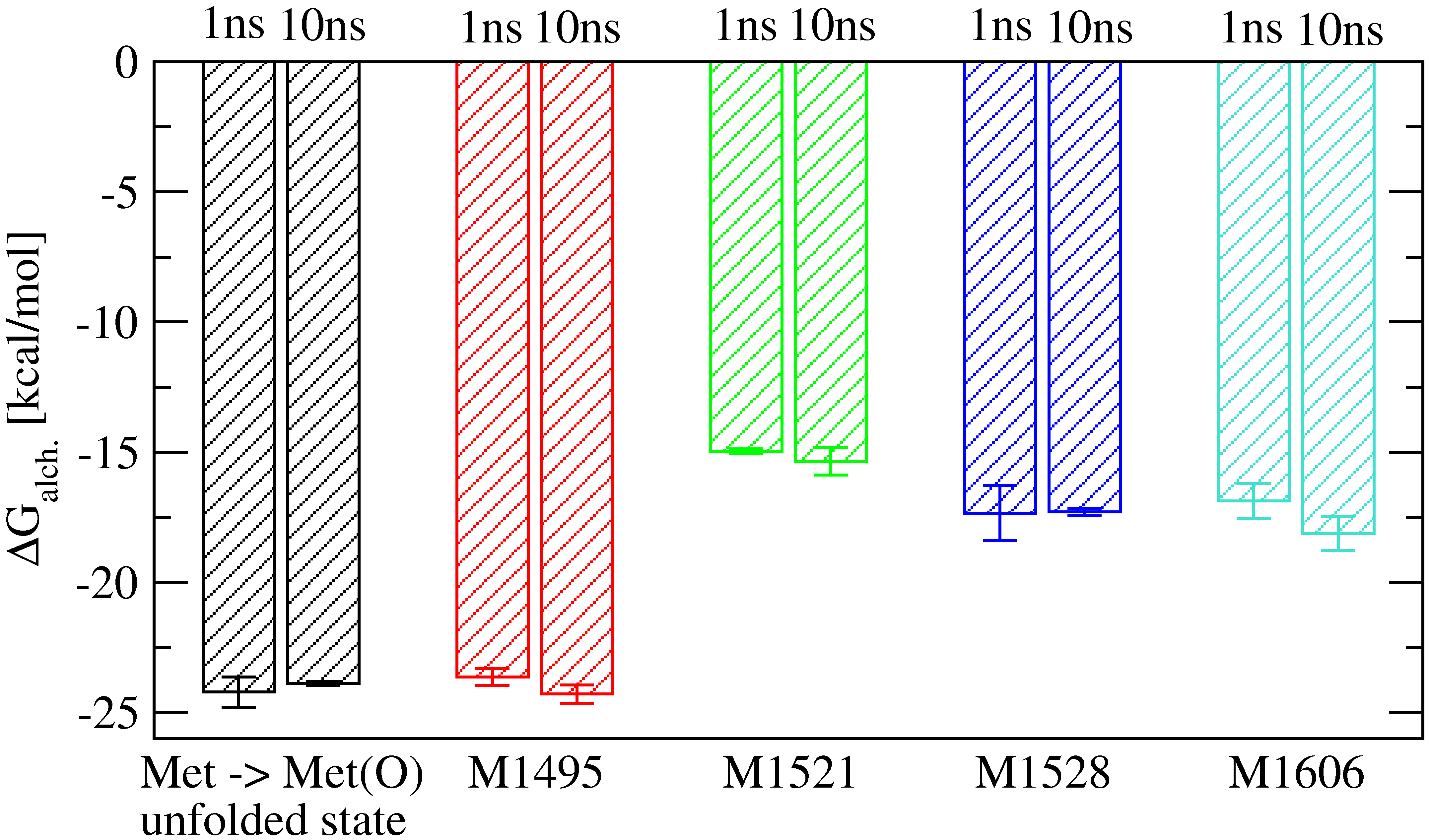

Supplement: S1 Fig — The reported values are averages over three simulations while error bars denote standard errors of the mean. (TIF) [file pone.0203675.s001.tif]

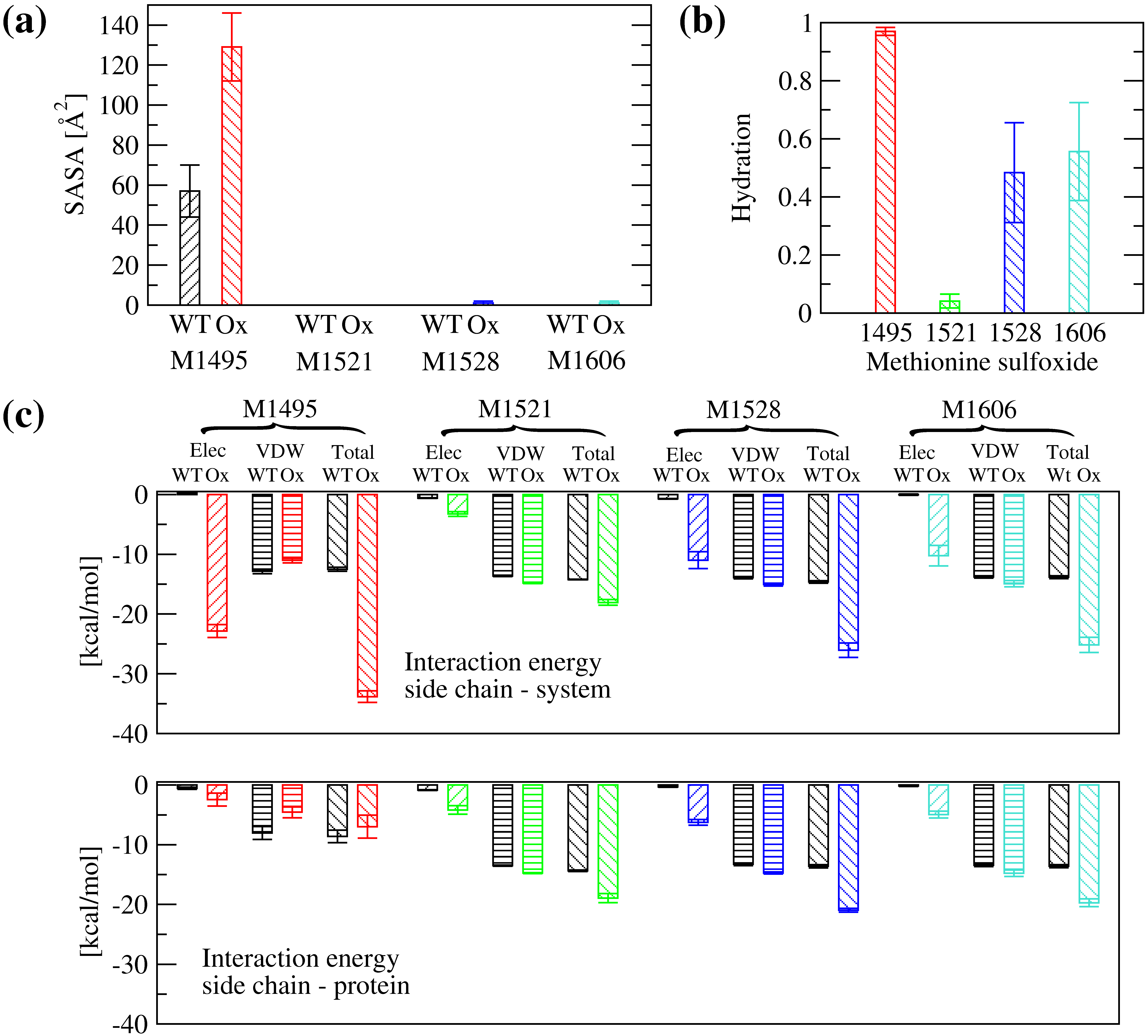

Supplement: S2 Fig — (a) SASA of methionine residues in the unoxidized (called here wild-type, WT) and the oxidized (Ox) state. (b) Fraction of frames where a methionine sulfoxide forms at least one hydrogen bond with water molecules. (c) Interaction energy between a methionine side chain and (top) the rest of the system or (bottom) the rest of the protein, respectively. Blue circles highlight significant electrostatic interactions between a residue and the solvent or the rest of the protein, respectively. Simulations were performed with the respective methionine residue in the oxidized state (runs M1495MO_1,2,3, M1521MO_1,2,3, M1528MO_1,2,3 and M1606MO_1,2,3 in Table 1) and compared in (a) and (c) to previously published runs with the unoxidized wild-type A2 domain [9]. The represented values are averages over three simulations while error bars denote standard errors of the mean. (TIF) [file pone.0203675.s002.tif]

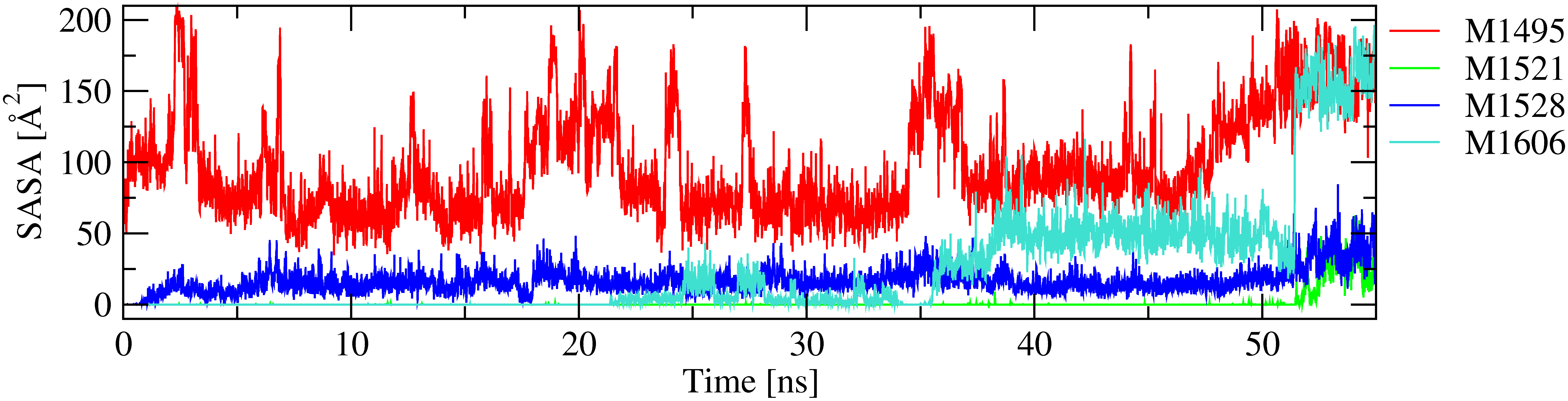

Supplement: S3 Fig — Presented here is an analysis of a previously published trajectory (run WT_pull_2) [9]. (TIF) [file pone.0203675.s003.tif]

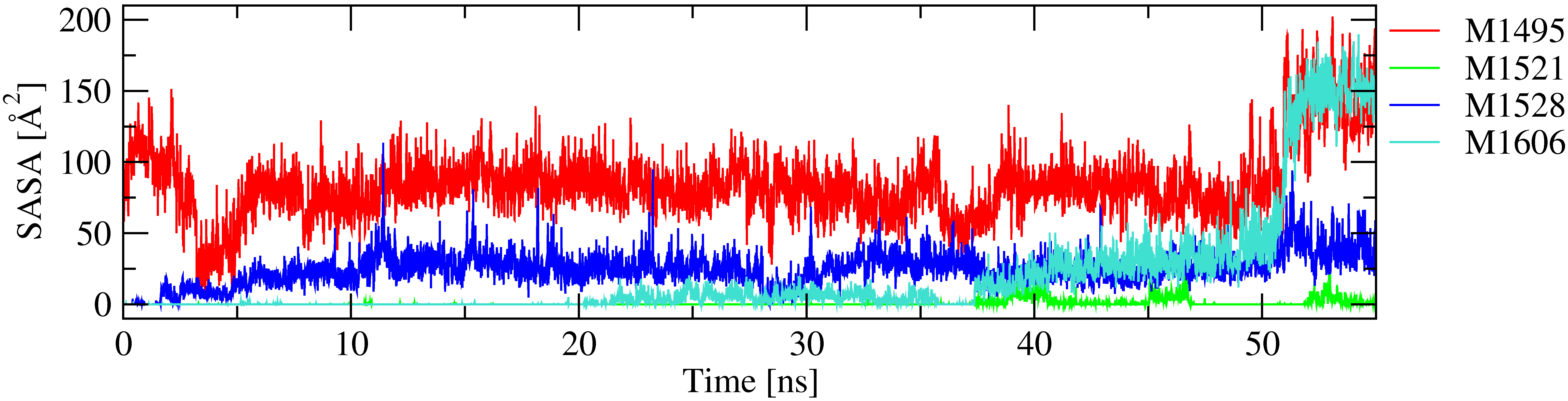

Supplement: S4 Fig — Presented here is an analysis of a previously published trajectory (run WT_pull_3) [9]. (TIF) [file pone.0203675.s004.tif]

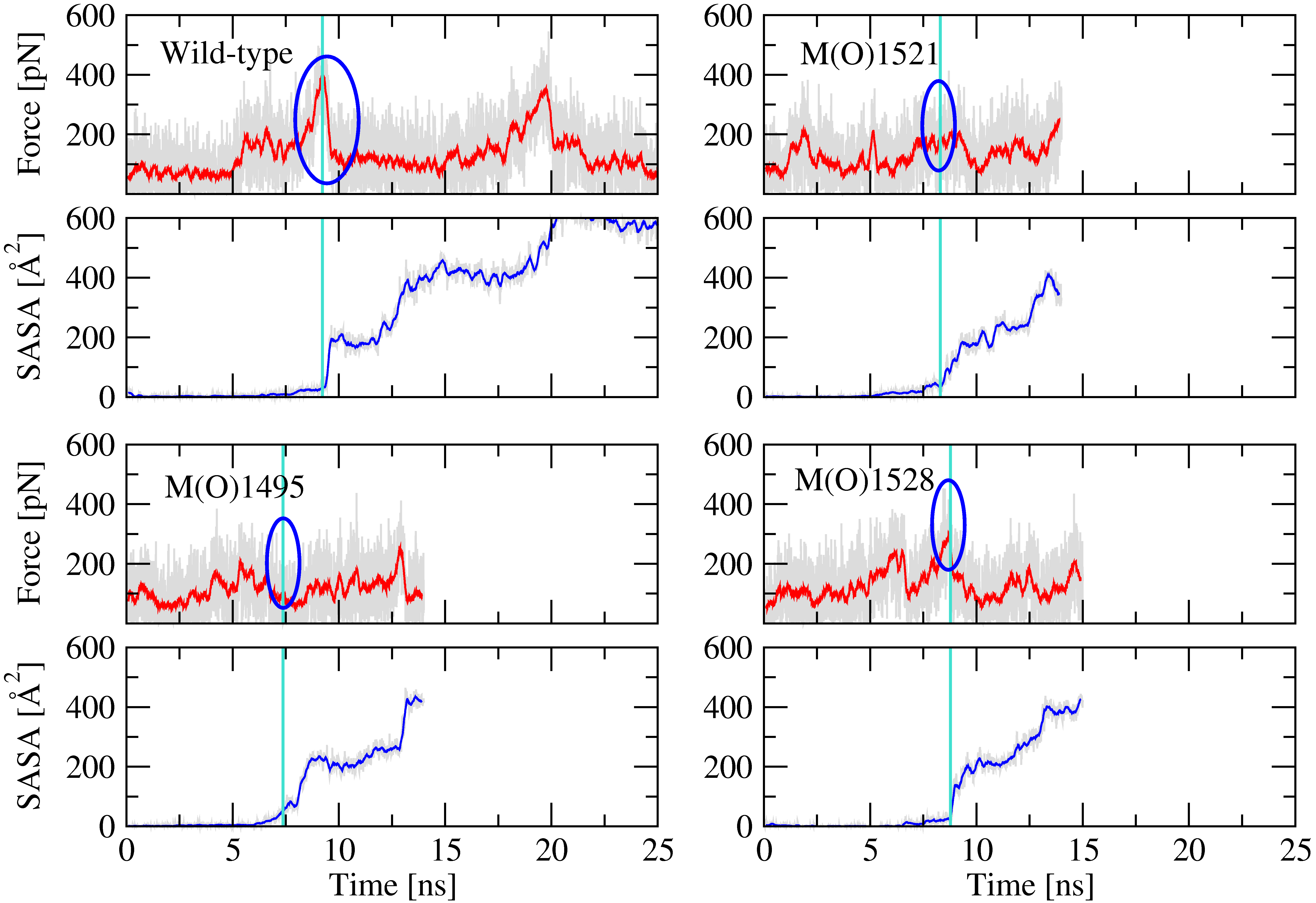

Supplement: S5 Fig — The vertical cyan line indicates the time point where the SASA exceeds 50 Å2. The blue circles highlight the corresponding peak in force. Running averages over 20 ps are indicated in red for the force and in blue for the SASA, respectively. The time series with the unoxidized with-type are taken from a previously published study (WT_pull_2) [9]. (TIF) [file pone.0203675.s005.tif]

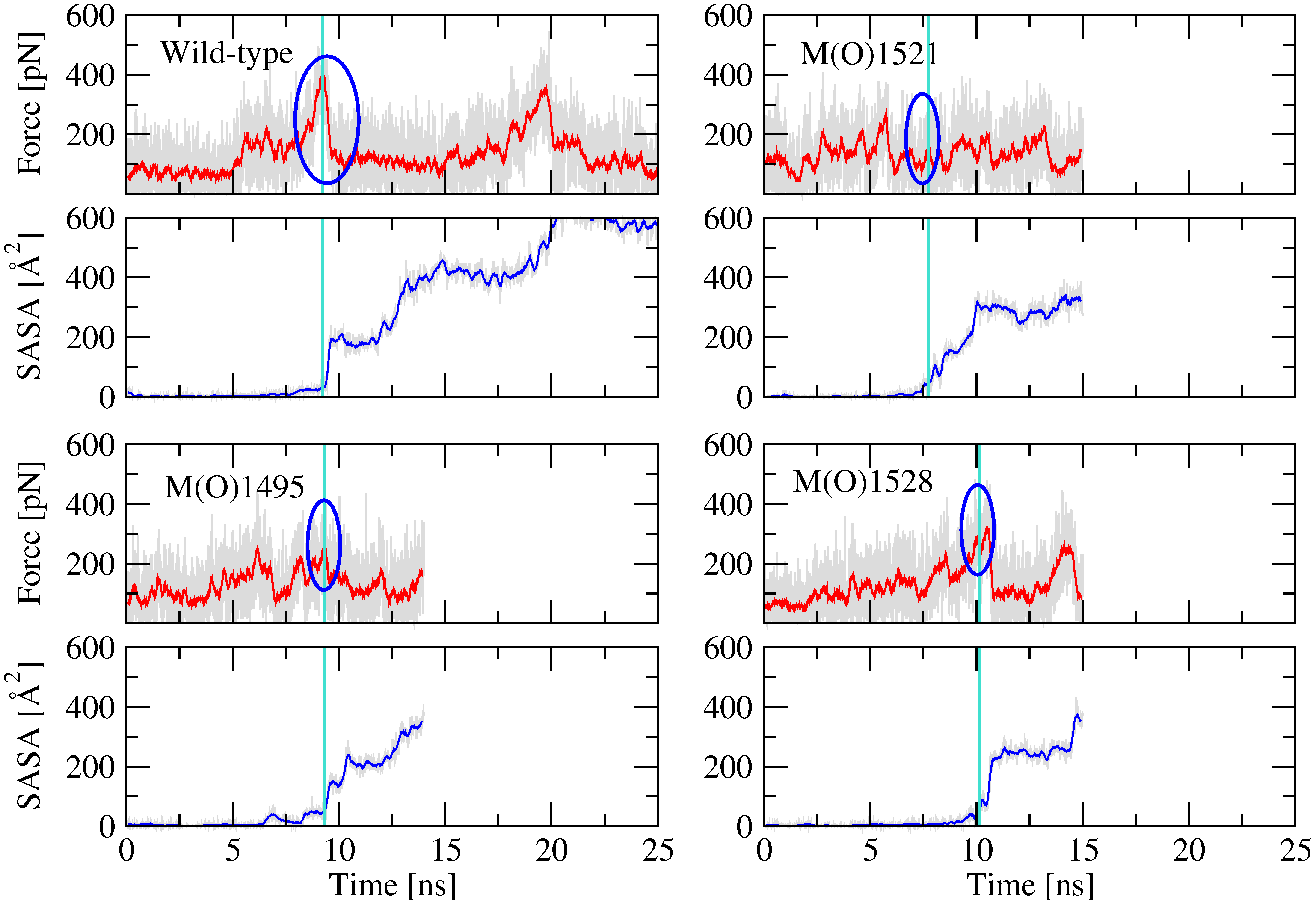

Supplement: S6 Fig — The vertical cyan line indicates the time point where the SASA exceeds 50 Å2. The blue circles highlight the corresponding peak in force. Running averages over 20 ps are indicated in red for the force and in blue for the SASA, respectively. The time series with the unoxidized with-type are taken from a previously published study (WT_pull_3) [9]. (TIF) [file pone.0203675.s006.tif]

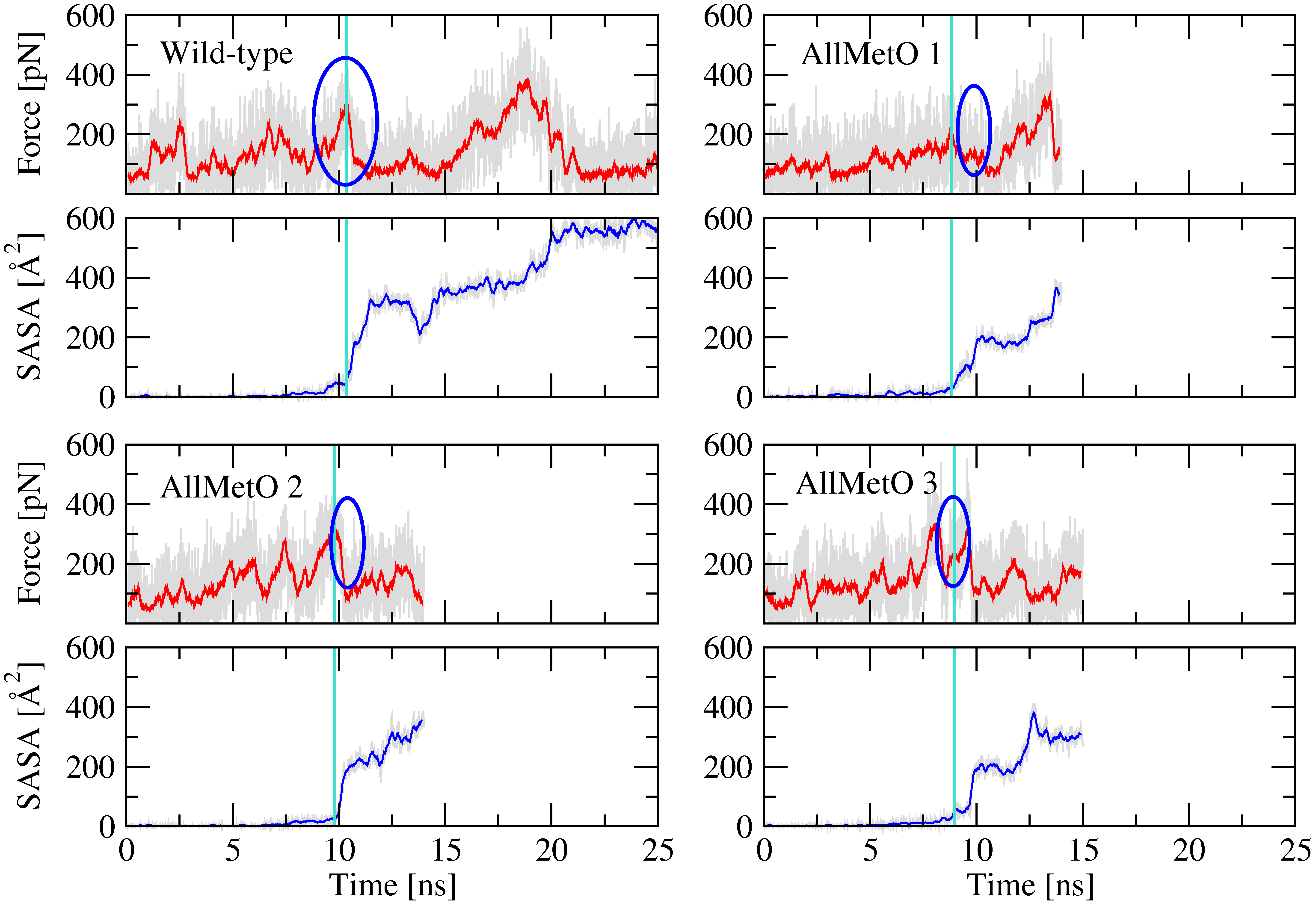

Supplement: S7 Fig — The vertical cyan line indicates the time point where the SASA exceeds 50 Å2. The blue circles highlight the corresponding peak in force. Running averages over 20 ps are indicated in red for the force and in blue for the SASA, respectively. The time series with the unoxidized with-type are taken from a previously published study (WT_pull_1) [9]. (TIF) [file pone.0203675.s007.tif]
